# Multinational, Multicenter Evaluation of Prostate Cancer Tissue in Sub-Saharan Africa: Challenges and Opportunities

**DOI:** 10.1200/GO.23.00403

**Published:** 2024-06-13

**Authors:** Abraham C. van Wyk, Priti Lal, J. Olufemi Ogunbiyi, Lynnette Kyokunda, Fred Hobenu, Cherif Dial, Mohamed Jalloh, Richard Gyasi, Olabode P. Oluwole, Afua D. Abrahams, Adam R. Botha, Nompumelelo Zamokuhle Mtshali, Caroline Andrews, Sunny Mante, Ben Adusei, Serigne M. Gueye, James E. Mensah, Andrew Anthony Adjei, Yao Tettey, Akin Adebiyi, Oseremen Aisuodionoe-Shadrach, Sefiu Bolarinwa Eniola, Amparo Serna, Kosj Yamoah, Wenlong Carl Chen, Pedro Fernandez, Brian D. Robinson, Juan Miguel Mosquera, Ann W. Hsing, Ilir Agalliu, Timothy R. Rebbeck

**Affiliations:** ^1^ Stellenbosch University and National Health Laboratory Service, Tygerberg Hospital, Cape Town, South Africa; ^2^ University of Pennsylvania, Philadelphia, PA; ^3^ University College Hospital/University of Ibadan, Ibadan, Nigeria; ^4^ University of Botswana, Gaborone, Botswana; ^5^ 37 Military Hospital, Accra, Ghana; ^6^ Hôpital Général Idrissa Pouye, Dakar, Sénégal; ^7^ Ecole Doctorale Universite Iba Der Thiam, Thiés, Sénégal; ^8^ Korle-Bu Teaching Hospital, Accra, Ghana; ^9^ University of Abuja, Abuja, Nigeria; ^10^ Cancer Science Centre, Abuja and University of Abuja Teaching Hospital, Abuja, Nigeria; ^11^ Department of Anatomical Pathology, Faculty of Health Sciences, University of the Witwatersrand and the National Health Laboratory Service, Johannesburg, South Africa; ^12^ Dana-Farber Cancer Institute, Boston, MA; ^13^ Moffitt Cancer Center & Research Institute, Tampa, FL; ^14^ National Cancer Registry, National Institute for Communicable Diseases a Division of the National Health Laboratory Service, Johannesburg, South Africa; ^15^ Strengthening Oncology Services Research Unit, Faculty of Health Sciences, University of the Witwatersrand, Johannesburg, South Africa; ^16^ Sydney Brenner Institute for Molecular Bioscience, Faculty of Health Sciences, University of the Witwatersrand, Johannesburg, South Africa; ^17^ Division of Urology, Department of Surgical Sciences, Stellenbosch University, Cape Town, South Africa; ^18^ Weill Cornell Medical Center, New York, NY; ^19^ Stanford Cancer Institute, Stanford School of Medicine, Palo Alto, CA; ^20^ Stanford Prevention Research Center, Stanford School of Medicine, Palo Alto, CA; ^21^ Albert Einstein College of Medicine, New York, NY

## Abstract

**PURPOSE:**

Prostate cancer disproportionately affects men of African descent, yet their representation in tissue-based studies is limited. This multinational, multicenter pilot study aims to establish the groundwork for collaborative research on prostate cancer in sub-Saharan Africa.

**METHODS:**

The Men of African Descent and Carcinoma of the Prostate network formed a pathologist working group representing eight institutions in five African countries. Formalin-fixed paraffin-embedded prostate tissue specimens were collected from Senegal, Nigeria, and Ghana. Histology slides were produced and digitally scanned. A central genitourinary pathologist (P.L.) and eight African general pathologists reviewed anonymized digital whole-slide images for International Society of Urological Pathology grade groups and other pathologic parameters. Discrepancies were re-evaluated, and consensus grading was assigned. A virtual training seminar on prostate cancer grading was followed by a second assessment on a subcohort of the same tissue set.

**RESULTS:**

Of 134 tissue blocks, 133 had evaluable tissue; 13 lacked cancer evidence, and four were of insufficient quality. Post-training, interobserver agreement for grade groups improved to 56%, with a median Cohen's quadratic weighted kappa of 0.83 (mean, 0.74), compared with an initial 46% agreement and a quadratic weighted kappa of 0.77. Interobserver agreement between African pathologist groups was 40%, with a quadratic weighted kappa of 0.66 (95% CI, 0.51 to 0.76). African pathologists tended to overgrade (36%) more frequently than undergrade (18%) compared with the reference genitourinary pathologist. Interobserver variability tended to worsen with a decrease in tissue quality.

**CONCLUSION:**

Tissue-based studies on prostate cancer in men of African descent are essential for a better understanding of this common disease. Standardized tissue handling protocols are crucial to ensure good tissue quality and data. The use of digital slide imaging can enhance collaboration among pathologists in multinational, multicenter studies.

## INTRODUCTION

### Prostate Cancer in Men of African Descent: A Global Health Challenge

Prostate cancer is a significant public health problem in men of African descent worldwide. In the United States, African American men (AAM) are more than twice as likely to die of prostate cancer compared with European American men.^1^ According to the GLOBOCAN 2020 estimates of cancer incidence and mortality, prostate cancer is the leading cancer in terms of both incidence and mortality among men in sub-Saharan Africa and the Caribbean.^2^ Prostate cancer rates in sub-Saharan Africa are potentially much higher than those reported in existing registries because of the absence of screening programs, limited cancer registry data, and limited access to health care.^3^ Members of the Men of African Descent and Carcinoma of the Prostate (MADCaP) network reported that prostate cancer prevalence in sub-Saharan Africa is comparable with that in AAM,^4^ who are believed to have some of the highest prostate cancer rates in the world.^5,6^

**CONTEXT**

**Key Objective**
Is it feasible to conduct multicenter, multinational tissue-based studies on prostate cancer in sub-Saharan Africa?
**Knowledge Generated**
Tissue-based studies are feasible within a collaborative network and are facilitated by digital slide imaging, but variability in tissue quality may pose challenges to interpretation by pathologists. Interobserver variability in prostate cancer grading tended to worsen with a decrease in tissue quality. In addition, the regulatory requirements of countries in Southern Africa for transferring human biologic material may cause delays.
**Relevance**
Standardized tissue handling protocols on the basis of best practices are crucial to ensure good tissue quality and reliable data. Sharing digital whole-slide images can effectively enhance collaboration among pathologists within a research network.

### Addressing the Knowledge Gap

Despite the evident public health implications of prostate cancer in men of African descent and the racial disparities in both prostate cancer incidence and mortality, our understanding of the underlying causes remains limited. This is, in part, due to the consistent under-representation of men with heritage other than European ancestry in prostate cancer studies.^7^

### The Urgency in Sub-Saharan Africa

A significant proportion of prostate cancer cases in sub-Saharan Africa are diagnosed at advanced stages with aggressive disease, often leading to incurable outcomes.^8^ Therefore, there is an urgent need to better understand prostate cancer in men of African descent. The knowledge gained from studying prostate cancer in sub-Saharan Africa can potentially improve our understanding of aggressive prostate cancer in men of African descent globally, including among AAM.

### Infrastructure Challenges and the Need for Research Capacity

Establishing infrastructure capable of addressing prostate cancer in sub-Saharan Africa will contribute to the development of research and clinical capacity. There is a general shortage of pathologists in sub-Saharan Africa. The majority of pathology services are concentrated in larger towns and cities, with little or no availability in rural areas.^9^ Similarly, there is limited formal assessment of the pathology evaluation practices commonly used for prostate tumor diagnosis in sub-Saharan Africa.

### Objectives of the Pilot Study

The primary objective of this pilot study was to assess the feasibility of conducting a multinational, multicenter prostate cancer tissue-based investigation in sub-Saharan Africa. The overarching goal was to establish the groundwork for a successful tissue-based prostate cancer project and to serve as a model for collaboration in tissue-based studies. Secondary objectives included enhancing our understanding of current pathology tissue handling practices at the MADCaP centers, formulating standardized protocols to bolster research capacity within the MADCaP network and to evaluate interobserver variability in the diagnosis and grading of prostate cancer among pathologists in our network. Concurrently, we aimed to assess the viability of sharing digital whole-slide pathology images within our group. Our intent was to identify challenges encountered during the pilot phase and devise strategies to address them.

## METHODS

### Study Sample and Tissue Collection

Eight African centers in Senegal (Hôpital Général Idrissa Pouye, Dakar), Ghana (37 Military Hospital and Korle-Bu Teaching Hospital, Accra), Nigeria (University College Hospital, Ibadan, and University of Abuja Teaching Hospital, Abuja), South Africa (Tygerberg Hospital, Cape Town and the University of the Witwatersrand, Johannesburg), and Botswana (University of Botswana, Gaborone) participated in sample accrual, evaluation, training, and resource development. Detailed descriptions of the MADCaP network have previously been reported.^10^

All patients seen in MADCaP clinics between 2015 and the end of 2019 who had a clinically or pathologically confirmed primary prostate cancer of any stage, grade, or pathologic classification were eligible to participate in this study. These were patients recruited for the genomics aspect of the study, and they had given consent for their tissue to be used in prostate cancer studies. Case status was confirmed by medical records review using a standardized abstraction form. For the purposes of this evaluation, tissue samples were collected from usual practice tissue diagnosis using the protocols applied individually at each center. The tissue was stored as formalin-fixed paraffin-embedded (FFPE) wax blocks according to the practices at each center. The data available for this study from the review of medical records of all study participants included basic demographics, cancer diagnosis, and epidemiologic risk factors.

Protocols for biosample sharing have been developed to allow samples to be sent without identifiers, but be linkable back to the original data set to allow for questions of quality control, additional data sharing for new analyses, and so on. Each center obtained local institutional review board approval for this study. The African centers were not subject to the Health Insurance Portability and Accountability Act of 1996, but these standards were imposed on the research conducted here. The data were held on a secure server using Federal Information Security Management Act guidelines and were not shared with other investigators within or outside the network without the express permission of the submitting center.

### Tissue Processing

We obtained FFPE prostate tissue samples for research purposes. Primary prostate tumor samples included prostate tissue obtained by needle biopsy, radical prostatectomy, or transurethral resection of the prostate (TURP) procedures from each center using the centers' usual practice protocols. For radical prostatectomy and TURP samples, the pathologist from each participating center was responsible for selecting one FFPE block most representative of the overall Gleason score/International Society of Urological Pathology (ISUP) grade group. The centers were instructed to submit an approximately equal number of cases from each ISUP grade group. Each center was required to maintain a screening log, which would enable tracking of the sample locally. For needle biopsy, the pathologists at the respective participating institutions selected one FFPE block representing the highest grade group. This selected biopsy was required to contain at least 1 mm total linear length of tumor tissue. If multiple blocks with similarly graded cancer existed, the pathologists were instructed to select the block with the highest percentage tumor involvement.

**FIG 1:**
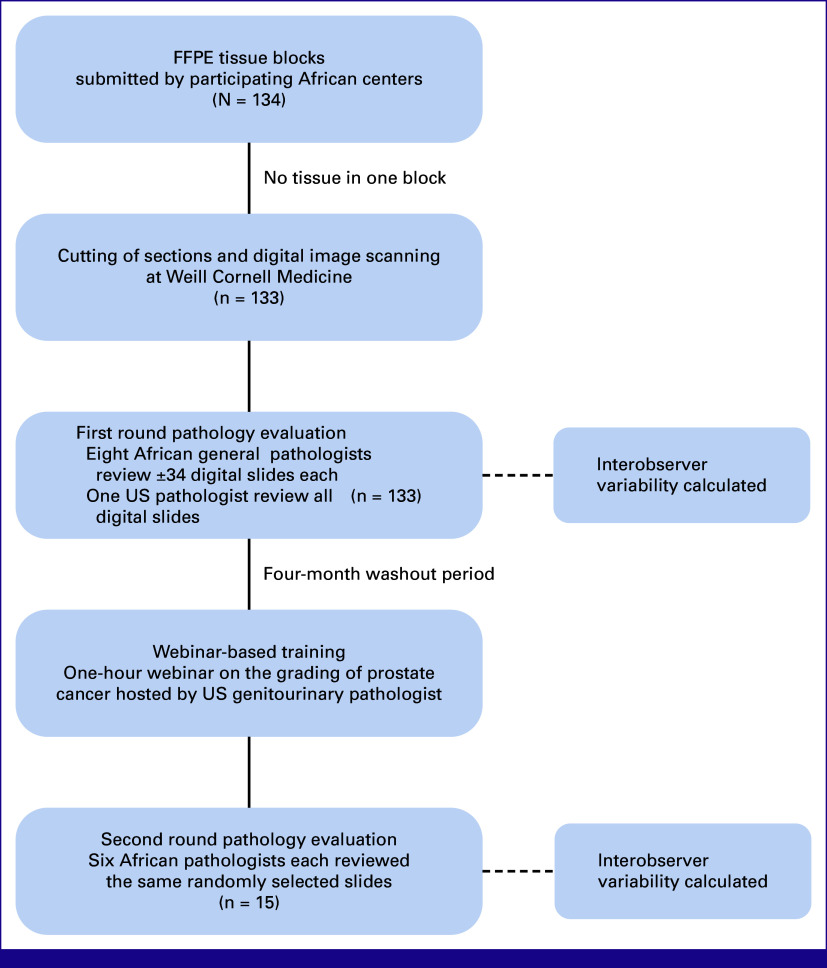
Study design. FFPE, formalin-fixed paraffin-embedded.

Participating African centers submitted FFPE tissue blocks for sectioning, staining, and digital scanning. The blocks were collected and shipped to the Dana-Farber Cancer Institute in Boston, MA, for central registration from where they were sent to the Weill Cornell Medical College (New York City, NY) for sectioning, staining, and whole-slide scanning. Tissue sections were cut at 3-5 µm. The hematoxylin and eosin (H&E)–stained slides were digitally scanned at 20× and uploaded to a central server. Figure 1 provides an overview of the study design.

### First Round Pathology Evaluation

The digitally scanned slides were randomly assigned to eight participating African pathologists, in such a way that each slide was independently evaluated twice by an African pathologist. A board-certified US genitourinary pathologist (P.L.) evaluated all digital slides. All participants used Aperio ImageScope (Leica Biosystems, Deer Park, IL), a free software package, to access and view the slides. The digital slides were assessed for the presence of cancer and, if detected, graded according to the 2014 ISUP consensus conference update on grading of prostate cancer.^11^ Additional pathologic parameters such as extraprostatic extension, perineural invasion, and the presence of cribriform pattern 4 were also evaluated. Pathologists had access only to the whole-slide image of an H&E section from the selected FFPE tissue block without the ability to examine deeper levels or use immunohistochemical stains for this evaluation. The pathology data were submitted to the Dana-Farber Cancer Institute in Boston, MA, for data storage.

After the first-round evaluation, cases with discordant grading were reviewed by P.L. and A.C.v.W. (a general anatomical pathologist from Africa), discussed online using the videoconferencing platform Zoom (Zoom Video Communications, Inc, San Jose, CA), and consensus grades were assigned. One of the pathologists (A.C.v.W.) rated the quality of the slides on the basis of the severity of artifact or other quality issues that could affect histopathologic interpretation, using a scale of 1 (no or mild effect on grading), 2 (moderate effect), and 3 (severe effect).

### Second Round Pathology Evaluation

After a washout period of 4 months, participating pathologists attended a 1-hour webinar presented by P.L. on prostate cancer grading. Subsequently, 15 digital slides were randomly selected from the original whole-slide image pool, shared, and independently re-evaluated by six African pathologists.

### Statistical Analysis

Deidentified data were captured electronically on a password-protected Microsoft Excel (2016) spreadsheet, and further statistical analysis was performed using Stata Statistical Software: Release 12 (Stata Corp, College Station, TX). Concordance among evaluators was assessed using percent agreement and Cohen's quadratic weighted kappa to measure inter-rater reliability. Missing variables were excluded from the statistical analysis.

## RESULTS

Of the eight MADCaP centers in sub-Saharan Africa that participated in the study, five centers located in West Africa (Nigeria, Ghana, and Senegal) contributed a total of 134 tissue specimens. Of the 134 tissue blocks submitted, 133 contained tissue suitable for preparing a histology slide.

### Clinical Descriptive Characteristics

The clinical descriptive characteristics and prostate-specific antigen (PSA) levels associated with the prostate tumor samples at the time of diagnosis are summarized in Table 1. The cohort showed remarkably high mean and median PSA levels of 943.3 ng/mL and 100 ng/mL, respectively, suggesting that a substantial number of patients likely had metastatic disease.

**TABLE 1:** Clinical Descriptive Characteristics of Prostate Tumor Samples With Obtainable Data at the Time of Diagnosis

Variable, No.	Evaluable Cases	Value	No. (%)
Country	133	Ghana	47 (35.3)
		Nigeria	61 (45.9)
		Senegal	25 (18.8)
Tumor location	97	Central zone	4 (4.1)
		Transitional zone	0 (0.0)
		Peripheral zone	29 (29.9)
		Multiple	31 (31.9)
		Not reported	33 (34.0)
Original Gleason score	110	6	32 (29.1)
		7	40 (36.4)
		8	22 (20.0)
		9	13 (11.8)
		10	3 (2.4)
Tumor stage	125	T1	12 (9.6)
		T2	49 (39.2)
		T3	54 (40.0)
		T4	10 (8.0)
		Not reported	1 (0.8)
Nodal status	48	NX	20 (41.7)
		N0	8 (16.7)
		N1	20 (41.7)
Metastasis	49	MX	13 (26.5)
		M0	9 (18.4)
		M1	15 (30.5)
		Not reported	12 (24.5)

Variable, No.	Evaluable Cases	Mean (median)	Range	SD
Age at diagnosis, years	133	69.1 (70)	50-99	9.1
PSA at diagnosis, ng/dL	109	943.3 (100)	7.7-23,959	3,062

NOTE. Evaluable cases refer to samples from which the variable could be obtained, from total n = 133 samples.

Abbreviations: PSA, prostate-specific antigen; SD, standard deviation.

### Pathologic Descriptive Characteristics

Of the 133 whole-slide digital images generated from the histology slides, 13 (9.8%) did not show any signs of cancer. Four (3.0%) had cancer, but the tissue quality was too poor to assign a grade, leading to their exclusion from the interobserver variability analysis. The pathologic descriptive characteristics of the prostate tumor samples after evaluating the digital images are summarized in Table 2. A significant proportion of patients in this group exhibited aggressive disease, as evidenced by the high frequencies of extraprostatic extension (7.5%), perineural invasion (30.8%), and cribriform pattern 4 (45%).

**TABLE 2:** Pathologic Descriptive Characteristics of Prostate Tumor Samples After Consensus Pathologic Evaluation

Variable, No.	Evaluable Cases	Value	No. (%)
Procedure by which tissue was obtained	133	Needle biopsy	123 (92.5)
		Radical prostatectomy	7 (5.3)
		TURP	3 (2.3)
Tumor present	133	Yes	120 (90.2)
		No	13 (9.8)
WHO grade group	120	1	18 (15)
		2	35 (29.2)
		3	17 (14.2)
		4	18 (15)
		5	28 (23.3)
		Unable to grade	4 (3.3)
Extraprostatic extension	120	Present	9 (7.5)
		Not demonstrated	111 (92.5)
Perineural invasion	120	Present	37 (30.8)
		Not demonstrated	83 (69.2)
Cribriform pattern 4	120	Present	54 (45)
		Not demonstrated	66 (55)
Glomeruloid pattern 4	120	Present	6 (5)
		Not demonstrated	114 (95)

Abbreviation: TURP, transurethral resection of the prostate.

### Participating Pathologists

The eight African pathologists were general anatomic pathologists from countries in West Africa (Senegal, Ghana, and Nigeria) and Southern Africa (South Africa and Botswana). The male-to-female ratio was 5 to 3, with an average age of 46.5 years (range, 40-60) and an average experience of 12.5 years postqualification as pathologists. The majority had no or limited experience with digital whole-slide imaging. A board-certified genitourinary pathologist from the United States served as the reference pathologist.

### Interobserver Variability

Table 3 presents the interobserver variability between the African pathologists and between the African pathologists and the US pathologist in assessing the presence or the absence of cancer and assigning a grade group. The interobserver agreement between African pathologists and the US pathologist improved from an initial agreement of around 46% (Cohen's quadratic weighted kappa: 0.77; 95% CI, 0.67 to 0.85) to 56.1% (Cohen's quadratic weighted kappa: median, 0.83; mean, 0.74) in the second round of evaluation. Compared with the US pathologist, African pathologists overgraded 36% and undergraded 18.2% of cases.

**TABLE 3:** Interobserver Variability in the First Round of Evaluation

Cancer Absent or Present (n = 129)	Agreement, %	Cohen's Quadratic Weighted Kappa (95% CI)
US *v* Afr1	95.0	0.72 (0.52 to 0.93)
US *v* Afr2	98.0	0.92 (0.81 to 1.00)
Afr1 *v* Afr2	93.8	0.66 (0.44 to 0.88)

WHO Grade Group (n = 129)^a^	Agreement, %	Cohen's Quadratic Weighted Kappa (95% CI)
US *v* Afr1	45.0	0.77 (0.68 to 0.84)
US *v* Afr2	46.5	0.77 (0.67 to 0.85)
Afr1 *v* Afr2	40.3	0.66 (0.51 to 0.76)

Abbreviations: Afr1, group 1 of the African general pathologists; Afr2, group 2 of the African general pathologists; US, US genitourinary pathologist.

^a^ Absence of cancer was graded as GG0.

### Tissue Quality and Its Effect on Interobserver Variability

A significant proportion of cases (69%) exhibited moderate to severe issues with tissue quality (Table 4). Inter-rater reliability tended to decrease as the tissue quality deteriorated. The most commonly encountered issues affecting histopathologic interpretation included fragmentation of tissue cores, multiple cores (>three) on one section, heat/drying artifact, trauma artifact, and apparently thick or overstained sections. Further investigation revealed that some of the tissue wax blocks melted during storage or transit, requiring the re-embedding of the tissue before imaging.

**TABLE 4:** Effect of Tissue Quality Score on Interobserver Variability

Quality Group	No. (%)	Raters	Agreement, %	Cohen's Quadratic Weighted Kappa	*P*
Q1	40 (31)	US *v* Afr1	55	0.76	<.001
		US *v* Afr2	63	0.88	<.001
		Afr1 *v* Afr2	48	0.78	<.001
Q2	71 (55)	US *v* Afr1	38	0.72	<.001
		US *v* Afr2	39	0.70	<.001
		Afr1 *v* Afr2	37	0.56	<.001
Q3	18 (14)	US *v* Afr1	50	0.77	<.001
		US *v* Afr2	39	0.51	.027
		Afr1 *v* Afr2	39	0.32	.174

Abbreviations: Afr1, group 1 of the African general pathologists; Afr2, group 2 of the African general pathologists; Q1, no or only mild quality issues; Q2, moderate quality issues; Q3, severe quality issues; US, US genitourinary pathologist.

## DISCUSSION

With this pilot study, we have demonstrated the feasibility of establishing a multicenter, multinational prostate tissue-based cancer study in a collaborative network in low- and middle-income countries in sub-Saharan Africa, by using digital slide imaging and videoconferencing. We have also identified several challenges and proposed strategies to address them.

Whole-slide imaging offered the opportunity to share pathology slides simultaneously among participating pathologists, eliminating the logistical challenges associated with sending physical glass slides. In addition, the use of videoconferencing software facilitated discussions between pathologists (P.L. and A.C.v.W.) regarding discordant cases although they were located in different continents. Looking ahead, the potential integration of image analysis by artificial intelligence may further optimize resources.

A large proportion of patients in this study exhibited indications of advanced and/or aggressive prostate cancer, as suggested by markedly elevated mean and median PSA levels and a high frequency of histopathologic parameters associated with aggressive disease. Extraprostatic extension, considered rare in prostate needle biopsies (prevalence of 0.6% in 19,950 patients^12^), was identified in 7.5% of samples in our predominantly needle biopsy–based study population (92.5%). Similarly, perineural invasion prevalence was high (30.8%) compared with a population-based study (16% prevalence^13^). It is essential to note a limitation: the generalizability of these histopathologic findings to prostate cancer in sub-Saharan Africa is uncertain because of the nonrandom nature of case collection in this study.

Several reasons account for the absence of cancer in 13 specimens (all core needle biopsies): potential loss during deeper study sections, inadvertent selection of incorrect tissue blocks, and the original diagnosis could have been incorrect, because of the unavailability of immunohistochemistry in some centers.

The challenges encountered in this study can be broadly described as regulatory difficulties, issues related to procedure standardization and quality control across centers, and interobserver variability in the grading of prostate cancer.

Of the eight centers that expressed their willingness to participate in this tissue pilot study, five (all situated in West Africa) were ultimately able to contribute cases. Unfortunately, the three centers in Southern Africa (two in South Africa and one in Botswana) were unable to contribute tissue because of delays in the preparation of material transfer agreements and tissue export permits. South Africa uniquely mandates the use of a standard material transfer agreement for tissue research by legislation, requiring parties outside the country to use South African material transfer agreements when transferring human biologic material to or from South Africa.^14^ The regulatory framework within which each research center operates should be taken into account when planning tissue research across countries. Sufficient time should be allocated to meet all regulatory requirements, but it is important to note that this can lead to increased financial and opportunity costs.

One of the best indicators of disease aggressiveness in prostate cancer is histopathologic grading, using Gleason score or grade group. Not only is it the most important predictor for biochemical recurrence, distant metastasis, and cancer-specific mortality in prostate cancer, but it is also the main stratification tool for deciding on different treatment options. Prostate cancer grading has been refined over the years, but its biggest shortcoming remains the notable interobserver variation among pathologists.^15^ Interobserver variation and periodic updates to the grading system make it difficult to directly compare Gleason scores between studies by different groups and those conducted during different time periods.^16^

**FIG 2:**
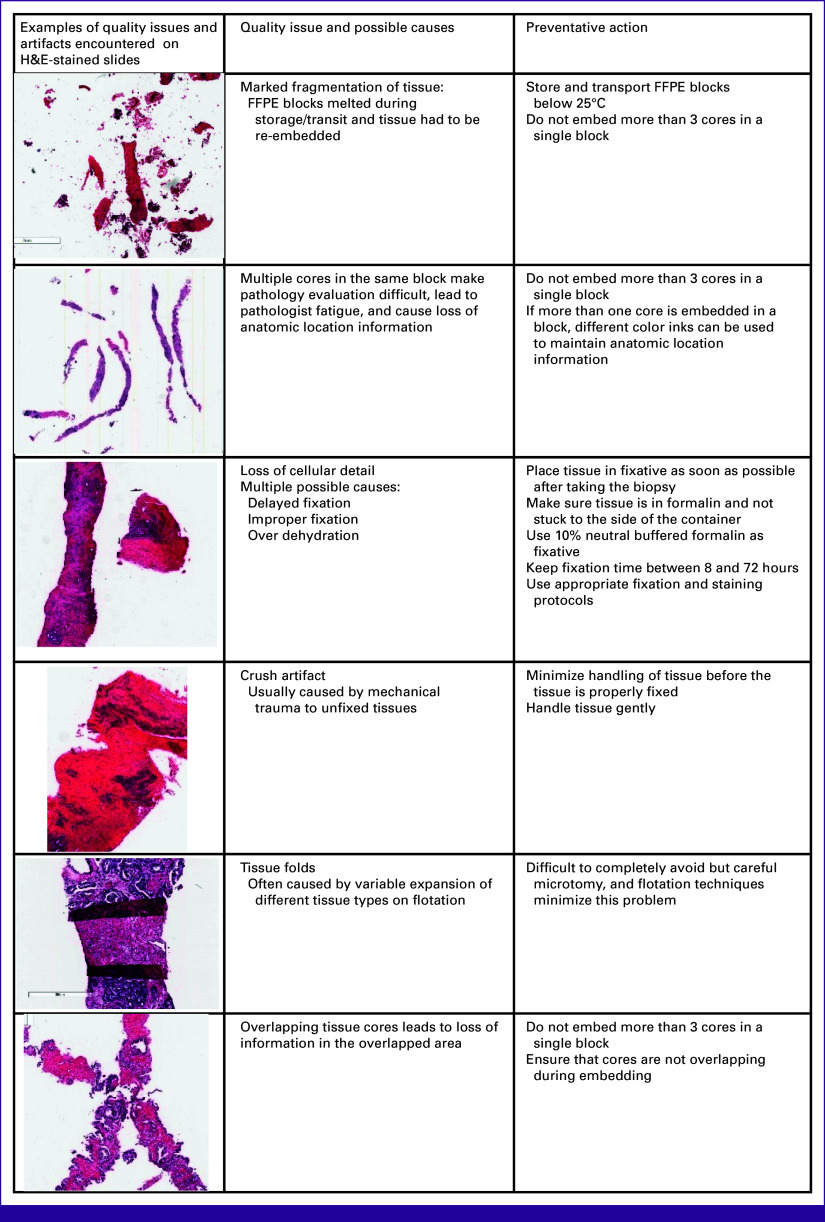
Quality issues and artifacts. FFPE, formalin-fixed paraffin-embedded; H&E, hematoxylin and eosin.

There are several possible reasons for the interobserver variability in this study, but in our opinion, a major contributing factor was the high frequency of tissue artifacts, which complicated histopathologic interpretation. A substantial portion (69%) of the whole-slide images exhibited features that hindered accurate pathologic review and interpretation (Fig 2). This was secondary to the quality of the tissue sections, rather than the process of digitally scanning the sections. Not surprisingly, the interobserver variability tended to worsen as the tissue quality deteriorated (Table 4). The correlation between poor slide quality and worsening interobserver variability was previously reported in diagnostic variability studies in breast pathology.^17^ Good tissue quality is not only essential for morphology-based studies but also crucial for other modalities by which tissue can be analyzed such as immunohistochemistry, fluorescence in situ hybridization, and DNA- and RNA-based tests.

Most of the tissue quality issues can be addressed through the implementation of standardized protocols and practices, from tissue fixation to processing, sectioning, and staining. Consequently, the MADCaP network has now incorporated these measures to ensure consistent histopathologic data for ongoing research.

Although it was not specifically investigated in this study, another factor that might have contributed to the interobserver variability was the use of digital slides in a setting where this is not a widely used format. Despite the obvious benefits as mentioned elsewhere in this article, a possible downside to their usage is a lack of familiarity with, and training in, digital slide interpretation, as well as a lack of standardization in the viewing equipment (monitor quality) used. Strategies for mitigation could include pathologist training, standardization of viewing equipment, and increased exposure to digital slides.^18^

Interobserver variability showed improvement in the second round of pathology evaluation after the educational webinar. Similar findings have been reported in other studies, where interventions such as educational lectures or tutorials,^19^ utilization of reference images,^20^ and review of general pathologist grading by genitourinary pathologists over a 1-year period^21^ have led to improved interobserver variability. With the integration of whole-slide imaging and videoconferencing platforms in our network, there is a great opportunity for increased interaction between general pathologists and genitourinary pathologists, ultimately aiming to improve interobserver variability.

Cases for this pilot study were selected in a nonrandom manner, restricting the generalizability of deductions that can be drawn from the clinical and histopathologic data. Only one specialist genitourinary pathologist was involved in the grading of prostate cancer cases, which served as the reference standard. The assessment of tissue quality was performed by a single pathologist. This inherently subjective evaluation may pose challenges in terms of reproducibility. The methodology used for the second round of pathology evaluations was not identical to the first round, posing challenges for direct comparisons.

In conclusion, a better understanding of prostate cancer in Africa not only would benefit men of African descent in Africa and the African diaspora but could also provide crucial insights into this common disease overall.^22^ To achieve this, more high-quality tissue-based studies on prostate cancer in men of African descent are needed, emphasizing consistent and standardized tissue handling protocols to ensure optimal tissue quality to facilitate the generation of robust and rigorous data. The use of digital slide imaging can enhance collaboration among pathologists internationally. Our experience aims to contribute to the establishment of tissue-based studies in low- and middle-income countries.
